# *Pediococcus acidilactici* CECT9879 (pA1c) Counteracts the Effect of a High-Glucose Exposure in *C. elegans* by Affecting the Insulin Signaling Pathway (IIS)

**DOI:** 10.3390/ijms23052689

**Published:** 2022-02-28

**Authors:** Deyan Yavorov-Dayliev, Fermín I. Milagro, Josune Ayo, María Oneca, Paula Aranaz

**Affiliations:** 1 Genbioma Aplicaciones SL. Polígono Industrial Noain-Esquiroz, Calle S, Nave 4, 31191 Esquíroz, Spain; deyan@genbioma.com (D.Y.-D.); josune@genbioma.com (J.A.); maria@genbioma.com (M.O.); 2 Center for Nutrition Research, Faculty of Pharmacy and Nutrition, University of Navarra, 31008 Pamplona, Spain; paranaz@unav.es; 3 Navarra Institute for Health Research (IdiSNA), 31008 Pamplona, Spain; 4 Centro de Investigación Biomédica en Red de la Fisiopatología de la Obesidad y Nutrición (CIBERObn), Instituto de Salud Carlos III, 28029 Madrid, Spain

**Keywords:** probiotic, diabetes, obesity, *Caenorhabditis elegans*, insulin-signaling-pathway, β-oxidation, *daf-16*

## Abstract

The increasing prevalence of metabolic syndrome-related diseases, including type-2 diabetes and obesity, makes it urgent to develop new alternative therapies, such as probiotics. In this study, we have used *Caenorhabditis elegans* under a high-glucose condition as a model to examine the potential probiotic activities of *Pediococcus* *acidilactici* CECT9879 (pA1c). The supplementation with pA1c reduced *C. elegans* fat accumulation in a nematode growth medium (NGM) and in a high-glucose (10 mM) NGM medium. Moreover, treatment with pA1c counteracted the effect of the high glucose by reducing reactive oxygen species by 20%, retarding the aging process and extending the nematode median survival (>2 days in comparison with untreated control worms). Gene expression analyses demonstrated that the probiotic metabolic syndrome-alleviating activities were mediated by modulation of the insulin/IGF-1 signaling pathway (IIS) through the reversion of the glucose-nuclear-localization of *daf-16* and the overexpression of *ins-6* and *daf-16* mediators, increased expression of fatty acid (FA) peroxisomal β-oxidation genes, and downregulation of FA biosynthesis key genes. Taken together, our data suggest that pA1c could be considered a potential probiotic strain for the prevention of the metabolic syndrome-related disturbances and highlight the use of *C. elegans* as an appropriate in vivo model for the study of the mechanisms underlying these diseases.

## 1. Introduction

Obesity is defined as a pathological state characterized by an excessive accumulation or abnormal distribution of body fat [1,2]. It is considered a chronic disease with a multifactorial origin that produce oxidative stress and a proinflammatory condition in the patient. This can lead to metabolic complications and the acceleration in the aging process [3,4,5]. Hence, obesity, together with other metabolic syndrome-related disturbances, like insulin resistance and type-2 diabetes, predispose one to suffer cardiovascular diseases [6,7,8,9].

The strategy to reduce the accumulation of fat and the excess of adipocytes in obese people consists of promoting a healthier lifestyle through diet, exercise or changes in behavior, together with the use of drugs, or in bariatric surgery [10,11]. Thus, there is no specific treatment against obesity and not all people respond in the same way to the treatment. For that reason, it has become urgent and necessary for the development of novel and alternative therapies to reduce the progression of this pathology, and at the same time, prevent metabolic syndrome-related diseases like type-2 diabetes.

During the last years it has been demonstrated the important role that gut microbiota plays on human health and the influence that alterations in this bacterial balance plays in the development of metabolic disorders [12]. Obesity is characterized by the imbalance of the microbiota composition (called dysbiosis), which activates the inflammatory pathways and contributes to the development of insulin resistance and diabetes [13,14]. Therefore, different bacterial strains have emerged as potential probiotics for the prevention of complications of these diseases, such as the excess of adiposity or the dysregulation of glycemia [15,16,17,18]. However, deeper investigations are needed to elucidate the effects of probiotic supplementation in human microbiota, since molecular mechanisms underlying their capacities are still unknown. One of these possible probiotic bacteria is *Pediococcus* *acidilactici* (PA). It is a species of Gram-positive coccus that is often found in pairs or tetrads. In addition, PA is a homofermentative bacterium that can grow in a wide range of pH, temperatures, and osmotic pressure, therefore being able to colonize the digestive tract. It has emerged as a potential probiotic that has shown promising results in animal and human experiments, though some of the results are limited. PA is commonly found in fermented vegetables, fermented dairy products, and meat.

Recently, *Caenorhabditis elegans* (*C. elegans*) has emerged as a novel experimental model for the study of lipid and carbohydrate metabolism, fat accumulation and health span [19,20]. Many of the genes involved in lipid regulation (intestinal function, fat metabolism and appetite) of *C. elegans* have been conserved in humans. Moreover, this nematode represents a reliable model to evaluate the effects of high glucose (trying to mimic overfeeding and diabetic conditions) on oxidative stress, aging, and lifespan. In this context, it has been established that a shortened lifespan in high glucose-exposed worms is in part due to the activation of the insulin/IGF-1 signaling (IIS) pathway [21]. Thus, *C. elegans* is considered as an appropriate initial screening in vivo model to evaluate the activity of different functional ingredients, including probiotic strains, on fat accumulation, glucose homeostasis, and lifespan, together with elucidating the mechanisms of action.

Previous studies in mice fed high fat and high sugar diets have demonstrated that *Pediococcus* *acidilactici* CECT9879 (pA1c) has beneficial properties on glucose homeostasis including an anti-hyperglycemic effects [22]. In this study, we have evaluated the metabolic activities of the probiotic strain *Pediococcus* *acidilactici* CECT9879 (pA1c) on *C. elegans* lipid accumulation, insulin/IGF-1 signaling (IIS), health span, oxidative stress, and aging, and its response to a high-glucose exposure. Gene expression analyses of different metabolic pathways, together with mutant strains of specific target genes, were assessed in order to shed light on the molecular mechanisms of action of this probiotic.

## 2. Results and Discussion

### 2.1. pA1c Reduces Fat Accumulation in C. elegans

**Figure 1:**
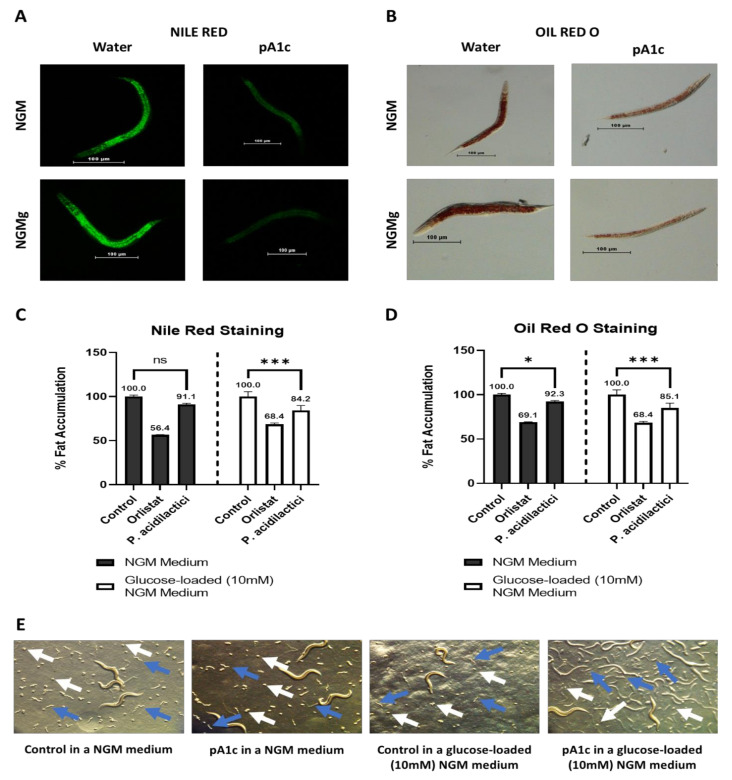
*Pediococcusacidilactici* reduces fat accumulation in *C. elegans* (**A**) Nile Red staining of water- and pA1c-treated worms in a NGM medium and in a glucose-loaded (10 mM) NGM medium. (**B**) Oil Red O staining of water- and pA1c-treated worms in a NGM medium and in a glucose-loaded (10 mM) NGM medium. (**C**) Nile Red quantification of water- and pA1c-treated worms (5 × 10^6^ CFU) in a NGM medium and in a glucose-loaded (10 mM) NGM medium. (**D**) Oil Red O quantification of water- and pA1c-treated worms (5 × 10^6^ CFU) in a NGM medium and in a glucose-loaded (10 mM) NGM medium. Results are expressed as the mean ± standard deviation relative to control worms in a NGM medium or in a glucose-loaded (10 mM) NGM medium. Significance refers to the effect of pA1c with respect to control worms in a NGM medium or in a glucose-loaded (10 mM) NGM medium (Student’s *t*-Test, * *p* < 0.05; *** *p* < 0.001). (**E**) Microscope observation of the presence of eggs (white arrows) and L1 larvae (blue arrows) in both water- and pA1c-supplemented plates in a NGM medium and in a glucose-loaded (10 mM) NGM medium. ns: not significant.

The measurement of fat storage and lipid droplets in C. elegans is possible by the visualization under microscopy of fat-soluble dyes such as Sudan Black B, ORO, and Nile Red [23,24]. In this case, two different stains were used to quantify fat accumulation: Nile Red (Figure 1A) and ORO (Figure 1B). It was observed that pA1c reduced fat accumulation in wild-type C. elegans in a NGM medium (8.8%) and in a glucose-loaded (10 mM) NGM medium (15.8%) with respect to control worms, quantified by Nile Red (Figure 1C). Similarly, pA1c reduced fat accumulation in a 7.7% in a NGM medium and in a 14.9% in a glucose-loaded (10 mM) NGM medium compared with control worms (Figure 1D), quantified by ORO. In both assays, the drug orlistat (1.5 mg/mL) was used as a positive control of fat reduction [25]. The fat-reducing activity of pA1c was independent of an effect on worm development. Comparing pA1c-treated worms with control worms, in plates of NGM medium with and without glucose, it was observed that both groups exhibited the presence of eggs (white arrows) and L1 larvae (blue arrows), with no differences in the time of appearance (Figure 1E).

There are many bioactive compounds capable of reducing the fat content in *C. elegans*. These components are well known and there are many studies that support their fat-reducing capacity. Some of them are phenolic compounds like resveratrol, curcumin, vanillic acid, or hesperidin [7,26,27,28,29,30]. Other compounds with body-fat reduction capacity are the free fatty acids (FAs) like omega-3 and omega-6 [31,32]. Although there are many works that demonstrate the fat-reducing properties of the bioactive compounds mentioned above, describing in detail their effects, there are very few studies testing the fat-reducing capacity of a probiotic in the *C. elegans* model. As far as we know, this is the first work that demonstrated the fat-reducing-capacity and potential mechanism of glucose regulation and of a pA1c strain, even of *Pediococcus* spp. in *C. elegans*. There are no previous works, or at least we have not seen any, in which a *Pediococcus* strain has reduced the fat accumulation in *C. elegans*. Nevertheless, other studies have found this fat-reducing effect in other bacterial strains in *C. elegans* [33,34,35], *Bifidobacterium* being the most relevant one.

Thus, we have demonstrated that supplementation with pA1c during larval stages reduces fat content without affecting the correct development of the worm. As *C. elegans* is not a very complex organism, its glucose and lipid metabolisms are very related and interconnected. Thus, it was observed that a more marked fat-reducing effect of the probiotic when glucose is present in the medium. This led us to the hypothesis that the insulin signaling pathway could be involved and it might have an important role in the mechanism of action of the probiotic. Therefore, our next step was the study of the glucose metabolism of the worm after the probiotic supplementation, with and without glucose in the medium.

### 2.2. pA1c Modulates the Insulin Signaling Pathway in C. elegans

Insulin/Insulin-like growth factor (IGF)-1 signaling pathway (IIS) is structurally and functionally conserved across evolution, from nematodes to mammals. Although there are many similarities between organisms, physiological differences in signaling still exist [36,37]. It is known that IIS in *C. elegans* is implicated in the regulation of glucose metabolism and diabetes, aging, and longevity [38,39,40], fat metabolism [35,41,42,43], stress resistance [38] (heat stress [44], oxidative stress [7,34,45], hypoxia [46], etc.) and behaviour [47]. The main components of this glucose-related metabolic pathway include the insulin-like peptides (ILP), which bind to the insulin/IGF-1 transmembrane receptor (IGFR) ortholog DAF-2 [36] activating it. This activation follows an activation of AGE-1 (*age-1* encodes a phosphatidylinositol-3-OH kinase (PI3K), which is a key upstream component of the IIS [48,49]), inducing the downregulation of DAF-16 and translocation to the nucleus by phosphorylation [49]. *C. elegans daf-16* encodes an ortholog of mammalian Forkhead box O transcription factor (FOXO) [50].

**Figure 2:**
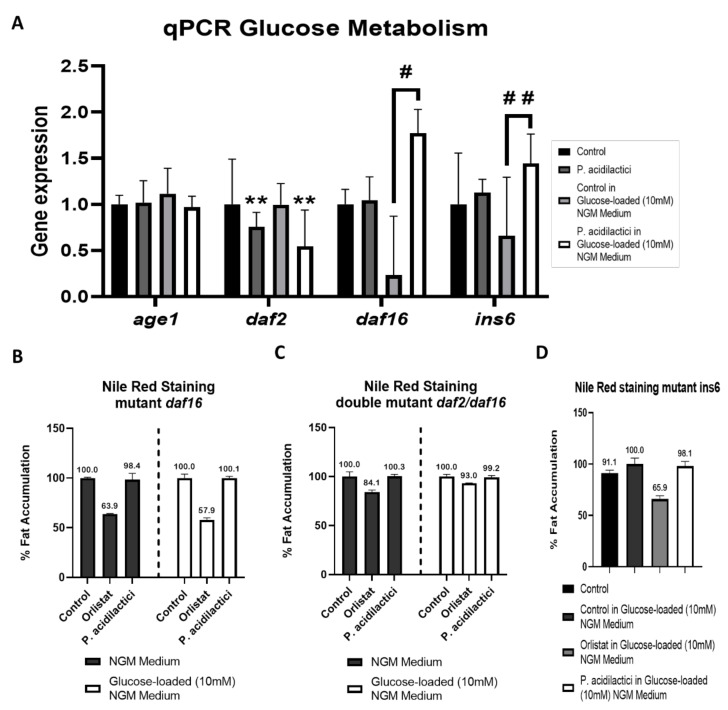
(**A**) Gene expression analysis quantified by real-time PCR (qPCR) in *C. elegans*. Gene expression levels were normalized to the housekeeping gene (*pmp-3*). Data are expressed using the 2^−∆∆Ct^ method. A two-way ANOVA (main effects: pA1c, glucose and their interaction), followed by a Student’s *t*-Test was carried out to evaluate statistical differences between groups: Two-way ANOVA results when pA1c factor is significant: ** *p* < 0.01; Student’s *t*-Test when interaction between factors is significant: # *p* < 0.05 vs. control in a glucose-loaded NGM medium, ## *p* < 0.01 vs. control in a glucose-loaded NGM medium. Nile Red quantification of PA-treated worms in *daf-16* mutant (**B**), double mutant *daf-2/daf-16* mutant (**C**) and *ins-6* mutant (**D**) in a NGM medium and in a glucose-loaded (10 mM) NGM medium. Statistical analyses were performed using Student’s *t*-Test to study differences between groups.

Our next step was the analysis of the main genes that encode the *C. elegans* IIS, to observe if the decrease in body fat was accompanied by changes in the glucose metabolism of the worm. The expression of the genes was quantified after pA1c supplementation in a medium without glucose, as in a medium with glucose (mimicking a diabetic worm model). Previous works showed that glucose-enriched conditions increase fat accumulation, upregulating IIS and reducing the expression of *daf-16* in *C. elegans* [35,48,51,52]. Our gene expression analyses demonstrated that the fat-reducing activity of the probiotic was mediated by the modulation of IIS. *ins-6* (an ILP), *age-1* and the key regulators of the IIS, *daf-2,* and *daf-16* were evaluated under normal condition and in a glucose-loaded medium. We observed a downregulation in the two upstream components of the IIS, *age-1,* and *daf-2* in the pA1c-treated worms, being only significant *daf-2*, and an upregulation of the ILP *ins-6* when glucose was in the medium. This was accompanied by an increase in the expression of *daf-16* in the pA1c-treated group in a glucose-loaded medium (Figure 2A). In agreement with the previously described works, we also observed that high-glucose augmented body fat by the upregulation of IIS and the inhibition of *daf-16*. On the contrary, supplementation with pA1c can reverse the fat accumulation induced by high-glucose by the downregulation of *daf-2*, and consequently by the upregulation of *daf-16*.

To shed light on the contribution of the IIS pathway in the pA1c-mediated fat reduction, *daf-16*, *daf-2/daf-16,* and *ins-6* mutants were analyzed after exposure to the probiotic. Nile Red staining revealed that the *daf-16* mutation overturns the pA1c-mediated body-fat-reducing effect (Figure 2B), demonstrating that the fat-reduction effect of pA1c is *daf-16* dependent. Also, it was observed that the supplementation with pA1c in the double mutant *daf-2/daf-16* (Figure 2C) and in the ILP *ins-6* mutant (Figure 2D) did not reduce the fat accumulation in *C. elegans*, suggesting that the *daf-16* modulation by the pA1c is also dependent on the insulin receptor (*daf-2*) and the ILP (*ins-6*). Hence, we can hypothesize that pA1c activity needs not only the *daf-16* effect, but all the insulin signaling pathway (IIS). These findings are consistent and support the data obtained from the study of pA1c supplementation in mice [22], in which it was seen that 12-week pA1c supplementation significantly attenuated body weight gain, mitigated glucose dysregulation by reducing fasting blood glucose levels, glucose tolerance test, leptin levels, and insulin resistance.

### 2.3. pA1c Inhibits the High-Glucose-Induced Nuclear Translocation of Daf-16

**Figure 3:**
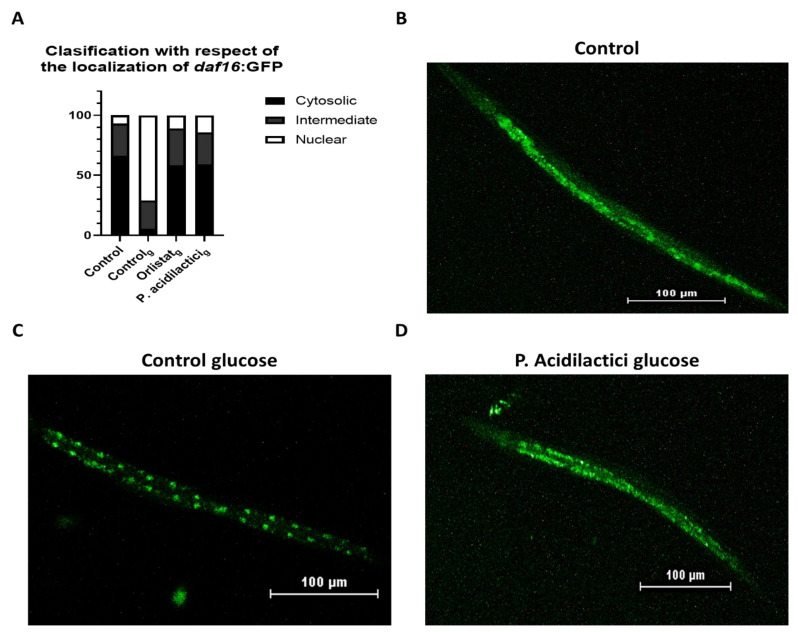
Classification of worms with respect of *daf-16:GFP* localization (**A**). Cytosolic *daf-16* expression of control worms in a NGM medium (**B**). The addition of glucose to the medium results in a nuclear localization of *daf-16* (**C**). Supplementation with pA1c reverts the *daf-16*-nuclear translocation effect of high-glucose and returns daf-16 into the cytosol (**D**).

As in mammals, under normal conditions insulin-like signaling appears to exclude DAF-16 from the nucleus in *C. elegans*. It is known that an impaired IIS by *age-1* or *daf-2* mutations results in *daf-16*-dependent increase in lifespan and stress resistance by its translocation to the nucleus [53,54]. However, no studies have found a link between the intracellular localization of *daf-16* and lipid metabolism. Thus, we found it interesting to analyze *daf-16* localization due to its high expression in pA1c-supplemented nematodes in a glucose-loaded medium. A high-glucose diet is associated with the development of metabolic diseases like obesity and type-2 diabetes, which decrease life expectancy; however, the mechanism through which a high-glucose diet (HGD) promotes these effects remains still unclear [55]. Previous studies considered HGD as a factor to develop osmotic and oxidative stress [56,57]. *daf-16* nuclear localization depends on many factors, including the modulation of IIS, the sensory perception of stimuli by the worm, and exposure to stress among others [53,54,57]. Therefore, contemplating HGD as an environmental factor that causes stress to the worms, we hypothesized that *daf-16* would transpose to the nucleus in response to stress conditions, such as the presence of glucose in the medium. Thus, while a higher cytosolic expression of *daf-16* was observed in control worms grown in NGM plates (Figure 3A,B), a nuclear-*daf-16*-translocation was observed when glucose (10 mM) was added into the medium (Figure 3A,C), confirming our hypothesis. However, pA1c-treated worms reverted the glucose-nuclear-localization of *daf-16* (Figure 3A,D). These findings would confirm the reducing effect of the probiotic on fat accumulation and demonstrates that pA1c is capable of reversing the effect of glucose on the IIS pathway mediator *daf-16*.

### 2.4. pA1c Modulates the Fatty Acid Metabolic Pathway in C. elegans

**Figure 4:**
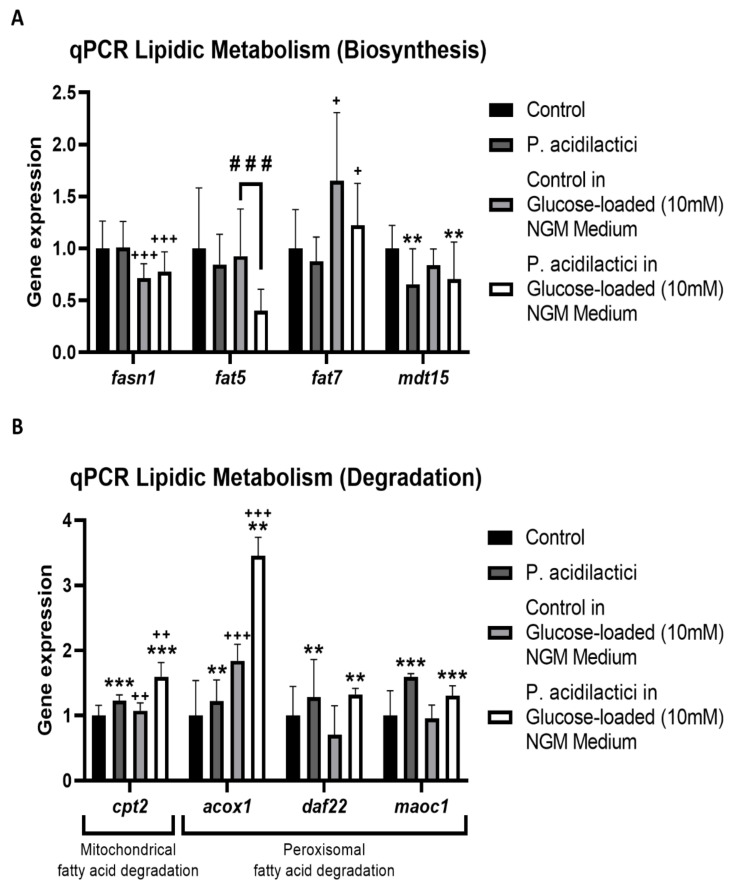
Gene expression analysis of key genes involved in the biosynthesis (**A**) and degradation (**B**) of lipids, quantified by real-time PCR (qPCR) in *C. elegans*. Gene expression levels were normalized to the housekeeping gene (*pmp-3*). Data are expressed using the 2^−∆∆Ct^ method. A two-way ANOVA (main effects: pA1c, glucose and their interaction), followed by a Student’s *t*-Test was carried out to evaluate statistical differences between groups: Two-way ANOVA results when pA1c factor is significant: ** *p* < 0.01, *** *p* < 0.001. Two-way ANOVA results when glucose factor is significant: + *p* < 0.05, ++ *p* < 0.01, +++ *p* < 0.001; Student’s *t*-Test when interaction between factors is significant: ### *p* < 0.001 vs. control in a glucose-loaded NGM medium.

Apart from the insulin signaling pathway, we wanted to analyze if any other metabolic pathway was involved in fat loss after pA1c supplementation. Fatty acids (FA) are carboxylic acids with long aliphatic chains, which in *C. elegans* lipids contain 14–20 carbons. FA are the building blocks and precursors for storage lipids (triacylglycerols), membrane lipids (phospholipids and sphingolipids), and signaling lipids (fatty acyl amides, eicosanoids, and others) [58,59,60]. Previous works have reported a reduction in fat accumulation and body fat in *C. elegans* by affecting the unsaturated FA biosynthesis [61,62,63] and FA degradation [5,31,32]. Here, it was evaluated as to if the fat-reducing activity of the probiotic was mediated by the FA metabolic pathway. Gene expression analyses showed an inhibition of the biosynthesis of FAs in the pA1c-treated groups, by the downregulation of *fasn-1* (encodes an ortholog of fatty acid synthase), *fat-5* (only significant in a glucose-loaded medium) and *fat-7* (two FAs-Δ9-desaturases) and *mdt-15* (a subunit of *C. elegans* mediator complex, SREBP- and NHR-49-interacting protein, and a transcriptional coactivator) (Figure 4A). *mdt-15* knockdown inhibits FA-biosynthesis by downregulating directly the FAs-Δ9-desaturases, *fat-5* and *fat-7*, decreasing the levels of unsaturated FA [64]. Therefore, we suggest that in addition to modulating the insulin pathway, the probiotic is involved in fatty acid biosynthesis, inhibiting it by acting directly on the *mdt-15* mediator complex, achieving a final downregulating effect on FAs-Δ9-desaturases, *fat-5* and *fat-7*, which was accompanied by a lower total fat accumulation. Moreover, β-oxidation gene expression analyses showed a significant upregulation in the groups supplemented with pA1c, with and without glucose in the medium, of both mitochondrial-FA degradation (*cpt-2*) and peroxisomal-FA degradation (*acox-1*, *daf-22* and *maoc-1*) (Figure 4B), suggesting an activation in the FA-degradation metabolism.

These results are in line with recent studies showing that flavanols [65], dihomo-gamma-linolenic acid [31], and other probiotics [35] were able to reduce fat accumulation in *C. elegans* through activation of the beta-oxidation process. Although there are studies reporting the reduction of fat accumulation by a probiotic through the beta-oxidation [34], this is the first work that reveals that a *P. acidilactici* strain is capable of modulating the beta-oxidation metabolic pathway in *C. elegans*.

Gene expression analyses quantified by quantitative-PCR analyses demonstrated that the fat-reducing activity of the probiotic was not only mediated by the modulation of the insulin/IGF-1 signaling (IIS) pathway, but also by an increased expression of genes involved in the fatty acid β-oxidation (mitochondrial and peroxisomal) and decreased expression of genes involved in fatty acid biosynthesis.

### 2.5. Supplementation with pA1c Enhanced Stress Oxidative Response by Reducing Reactive Oxygen Species (ROS), Improved Aging and Increased Lifespan in C. elegans

The obtained results about the fat-reduction activity of the probiotic, together with the modulation of the insulin signaling pathway, led us to continue investigating other metabolic pathways that the probiotic could be affecting. We focused on the health span of the worm since fat reduction is usually accompanied by an improvement of oxidative stress response, aging, or *C. elegans* lifespan.

In homeostatic conditions, redox reactions are kept in balance. Oxidative stress is a result of an imbalance in this equilibrium due to excessive increase in oxidizers, occurring when the antioxidant systems are not able to neutralize the ROS produced by the oxidants [66]. It has been described that different probiotic (specially *Lactobacillus* [67,68,69] and *Bifidobacterium* [70]) and bioactive compounds, such as curcumin [71], resveratrol [5,72,73,74], and apigenin [5] improve stress resistance response. In this work, the oxidative stress-alleviating properties of pA1c were analyzed. Fluorescent dye dihydroethidium (DHE) was used to quantify ROS levels in vivo.

**Figure 5:**
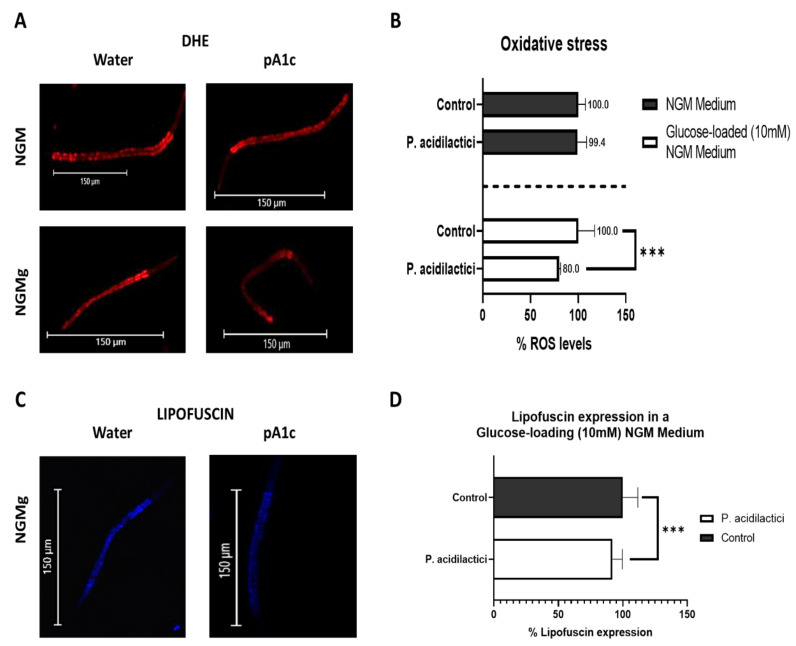
*Pediococcusacidilactici* improves *C. elegans* health span (**A**) DHE staining of water-and pA1c-treated worms in a NGM medium and in a glucose-loaded (10 mM) NGM medium. (**B**) ROS production quantification (measured by DHE) of water- and pA1c-treated worms (5 × 10^6^ CFU) in a NGM medium and in a glucose-loaded (10 mM) NGM medium. Results are expressed as the mean ± standard deviation relative to control worms in a NGM medium or in a glucose-loaded (10 mM) NGM medium. Significance refers to the effect of pA1c with respect to control worms in a NGM medium or in a glucose-loaded (10 mM) NGM medium (Student’s *t*-Test, *** *p* < 0.001). (**C**) Lipofuscin aging pigment of water-and pA1c-treated worms in a glucose-loaded (10 mM) NGM medium. (**D**) Quantification of lipofuscin aging pigment of water- and pA1c-treated worms (5 × 10^6^ CFU) in a glucose-loaded (10 mM) NGM medium. Results are expressed as the mean ± standard deviation relative to control worms. Significance refers to the effect of pA1c with respect to control worms in a NGM medium or in a glucose-loaded (10 mM) NGM medium (Student’s *t*-Test, *** *p* < 0.001).

The treatment with pA1c during the larval stages improved *C. elegans* health span. pA1c induced the oxidative stress response, but only when glucose was added to the medium, reducing ROS levels in a 20% compared with control worms (Figure 5A,B). The lack of effect of the probiotic in a non-glucose medium could be explained by the fact that in a medium without glucose the worms are not exposed to a high stress stimulus. On the other hand, as explained above, glucose itself acts as an environmental factor that induces stress to the worms, hence, in a medium with glucose, pA1c reverts the stress-effect originated by glucose.

Aging is defined as the accumulation of diverse deleterious changes occurring in cells and tissues with advancing age that are responsible for the increased risk of diseases and death [75,76]. Age is the main risk factor for common diseases in developed countries, such as type-2 diabetes, cancer, cardiovascular disease, and neurodegeneration [75,77,78].

In recent years, a number of probiotics and bioactive compounds have been reported to modulate aging and to increase lifespan in *C. elegans*. For example, supplementation with *Butyricicoccus* *pullicaecorum* increases the lifespan and retards aging in *C. elegans* via the transforming growth factor-beta (TGF-β) pathway associated with anti-inflammatory processes in the innate immune system [79]; *Propionibacterium* extends the mean lifespan and retards aging in *C. elegans* via activation of the innate immune system [80]; *Clostridium butyricum* MIYAIRI 588 increases the lifespan in *C. elegans* through regulation of the IIS and the Nrf2 transcription factor [81]; *Lactobacillus rhamnosus* CNCM I-3690 strain increases average worm lifespan by 20% by affecting the IIS in *C. elegans* [69]. One of the proposed mechanisms for the lifespan extension could be that the colonization of the *C. elegans* intestines by the probiotic strains would affect the metabolization of the nematode diet, as suggested by Shaikhulova et al. [82].

Regarding bioactive compounds, the treatment with caffeine extends lifespan by a DAF-16/FOXO-independent pathway in *C. elegans* [83]; calycosin (a naturally occurring flavonoid extracted from *Mongholicus* *Bunge*) promotes lifespan in *C. elegans* through IIS via *daf-16*, *age-1* and *daf-2* [84]; D-glucosamine (an amino sugar) supplementation extends life span of nematodes by impairing glucose metabolism that activates AMP-activated protein kinase (AMPK/AAK-2) and increases mitochondrial biogenesis [85]; diosgenin, a phytosterol, prolongs the lifespan and mitigates glucose toxicity via DAF-16/FOXO and GST-4 in *C. elegans* [86].

Lipofuscin pigment autofluorescence, present in all worms, was set as an aging marker. Here, this parameter was used to estimate aging in *C. elegans*. Treatment with pA1c from larval stage-1 to larval stage-4 caused a significant reduction (10%) of the lipofuscin aging pigment in comparison with the control worms. As in the case of oxidative stress, this reduction was only observed in a glucose-loaded medium (Figure 5C,D).

**Figure 6:**
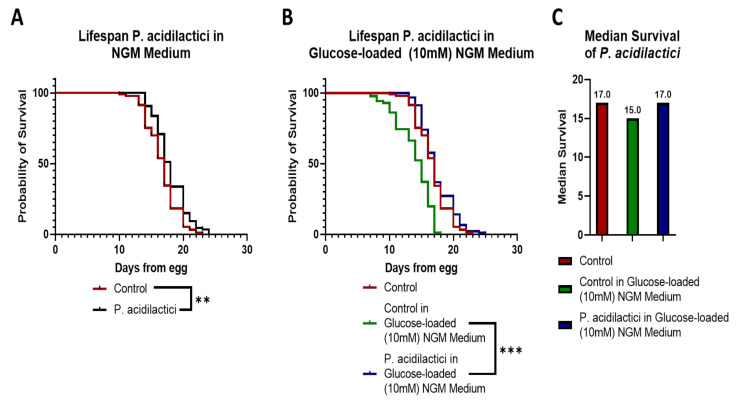
*Pediococcusacidilactici* increases lifespan of *C. elegans* (**A**) Lifespan analyses of water- and pA1c-treated worms in a NGM medium. Significance refers to the effect of pA1c with respect to control worms in a NGM medium (log-rank Mantel-Cox Test, ** *p* < 0.01). (**B**) Lifespan analyses of water- and pA1c-treated worms in a glucose-loaded (10 mM) NGM medium. Significance refers to the effect of pA1c with respect to control worms in a glucose-loaded (10 mM) NGM medium (log-rank Mantel-Cox Test, *** *p* < 0.001). (**C**) Median survival (in days) of control worms in a NGM medium and in a glucose-loaded (10 mM) NGM medium, and of pA1c-treated worms in a glucose-loaded (10 mM) NGM medium.

In addition, pA1c induced a significant increase in the worm lifespan. pA1c treatment increased the lifespan of the nematode in one day in a NGM medium compared with control worms (Figure 6A). Moreover, when glucose was added to the medium, the probiotic augmented its effect and increased *C. elegans* lifespan in two days compared with control worms in a glucose-loaded medium (Figure 6B). Glucose is known to reduce lifespan in *C. elegans*, and it was observed that pA1c lengthens lifespan more when glucose is present in the medium. Therefore, we can hypothesize that the probiotic reverts the glucose-induced shortening effect on lifespan. In fact, we observed that nematodes treated with pA1c in a glucose-loaded medium exhibit similar values of median survival than control worms in a normal NGM medium (Figure 6C).

As we observed in the studies on aging and lifespan, other probiotics and bioactive compounds are able to modulate the life expectancy and stress response of the worm through the insulin signaling pathway, specially the FOXO-transcription factor DAF-16, which is known to be involved in several physiological functions like aging, development, fat accumulation, stress and metabolism [87]. For example, blueberry increased the lifespan, decreased lipofuscin accumulation, improved health indexes, and enhanced stress resistance in *C. elegans* via DAF-16 [88], showing the important role of the IIS in all biological functions. Although no obvious nuclear translocation of *daf-16* was observed under pA1c supplementation (Figure 3), pA1c-treatment increased mRNA expression of *daf-16* (Figure 2A). Moreover, pA1c inhibited the expression level of the genes upstream of *daf-16* in the IIS (*daf-2* and *age-1*, Figure 2A). Besides IIS, it has been shown that NHR-49/PPAR-alpha is also involved in the regulation of lifespan in *C. elegans* [89,90]. In addition, SKN-1/Nrf2 plays an important role in wide range of homeostatic functions, one of them is the life extension of the worm by inducing the stress response and therefore decreasing intracellular ROS levels [89,91,92,93].

**Figure 7:**
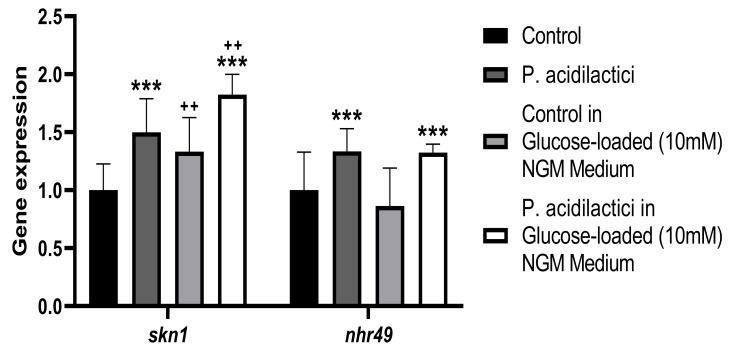
Gene expression analysis quantified by real-time PCR (qPCR) in *C. elegans*. Gene expression levels were normalized to the housekeeping gene (*pmp-3*). Data are expressed using the 2^−∆∆Ct^ method. A two-way ANOVA (main effects: pA1c, glucose and their interaction), followed by a Student’s *t*-Test was carried out to evaluate statistical differences between groups: Two-way ANOVA results when pA1c factor is significant: *** *p* < 0.001. Two-way ANOVA results when glucose factor is significant: ++ *p* < 0.01.

Gene expression analyses confirmed the PA-induced improvement of health span parameters, like the increase of the oxidative stress response by the upregulation of *skn-1* and the reduction of ROS levels, and the increase of lifespan expectancy by the upregulation of *nhr-49* (Figure 7), in both NGM and glucose-loaded plates.

These results suggest that pA1c ameliorates aging (reducing lipofuscin pigment), enhances stress-oxidative response by the modulation of the SKN-1/Nrf2 metabolic pathway, and extends the lifespan of *C. elegans* by regulating the IIS and the NHR-49/PPAR-Alpha pathway. As in the case of fat accumulation and the activation of beta oxidation, this is the first study that demonstrates that this pA1c strain ameliorates oxidative stress response (reducing ROS), improves aging, and increases lifespan in high-glucose-exposed *C. elegans*.

## 3. Matherial and Methods

### 3.1. Strains and Culture

The bacterial strain worked with in this study was *Pediococcus* *acidilactici* CECT9879 (pA1c), provided by Genbioma Aplicaciones S.L. (Polígono industrial Noain-Esquiroz, S Street, Nave 4, Navarra, 31191, Spain). pA1c was grown in deMan-Rogosa-Sharpe Agar (MRS) medium at 37 °C (facultative anaerobe). pA1c was used at a concentration of 5 × 10^6^ CFU/mL for all the assays. The *C. elegans* strains used were: N2 Bristol as wild-type strain, *daf-16* (mu86, CF1038) mutant strain, *daf-2/daf-16* (GR1308) double mutant strain, *daf-16:GFP* mutant strain and hpDf761 II (hpDf761 removes *ins-4*, *ins-5*, and *ins-6*) mutant strain. All strains were obtained from the Caenorhabditis Genetics Center (CGC, University of Minnesota, Minneapolis, MN, USA). *C. elegans* was cultured on nematode-growth-medium (NGM) at 20 °C. *Escherichia coli* OP50 (*E. coli*, grown in LB Broth Lennox at 37 °C) was utilized as standard nematode sustenance.

### 3.2. Experimental Design

All tests were carried out in quadruplicate, in 6-well cell culture plates with 4 mL NGM or with 4 mL glucose-loading (10 mM) NGM (NGMg) per well (with the pA1c spread or not inside the medium). Plates with Orlistat (1.5 mg/mL, Sigma Aldrich, St. Louis, MO, USA) were used as positive control of fat accumulation reduction. All experiments were performed at the concentration of 5 × 10^6^ CFU/mL (colony-forming unit/milliliter of water) of pA1c. After pA1c were added to NGM, plates were allowed to solidify and dry in the dark to protect them from light oxidation. Thereafter, 150 μL of an overnight culture of *E. coli* OP50 were seeded, and plates were again incubated until dried at room temperature in the dark. For all assays, gravid animals were subjected to a standard hypochlorite treatment to obtain age-synchronized worms (wild-type or mutants). The eggs were allowed to hatch overnight in M9 medium and about 2000 L1 larvae were transferred onto plates and grown until L4 or one-day adult stage.

### 3.3. Nile Red Staining

Nile Red staining is a dye for neutral lipids. Briefly, L4 worms grown in NGM or NGMg with different treatments were collected in 1.5 mL tubes and washed three times with PBST (0.01% of Triton X-100 in phosphate buffered saline). Then, the worms were put on ice for 15 min and fixed in 40% isopropanol for 3 min. Staining was carried out by adding 150 μL of Nile Red solution (3 μg/mL) per tube and incubating (30 min) with moderate shacking at room temperature in the dark. After that, the worms were washed in PBST and mounted on a 2% agarose pad for microscopy visualization.

### 3.4. Oil Red O (ORO) Staining

For ORO staining, the worms harvested as previously explained were fixed in 60% isopropanol for 5 min. ORO solution was prepared the day before use by diluting stock (0.5% ORO in isopropanol) to 60% with water, filtered (0.45 μm), stirred at room temperature overnight and filtered again just before use. Samples were incubated in this solution for 6 h in a wet chamber with gentle rocking in the dark, washed, and stored in PBST at 4 °C until visualization.

### 3.5. DHE Staining

Fluorescent dye dihydroethidium (DHE; Dihydroethidium BioReagent, ≥95% (HPCE), Sigma-Aldrich, St. Louis, MO, USA) was used to quantify ROS levels in vivo. Briefly, 750 synchronized L1 larvae were transferred into NGM or NGMg plates holding water (control group) or pA1c and were allowed to grow in them until they reached L4. Once they reached that stage, L4 worms were harvested, washed with PBST, and incubated in a 3 μM DHE solution (in PBS) during 30 min. Subsequently, worms were washed with PBST and mounted on a 2% agarose pads with a 1% of sodium azide.

### 3.6. C. elegans Aging Visualization

Lipofuscin pigment autofluorescence, present in all worms, was set on as an aging marker. This parameter was utilized to estimate aging in *C. elegans*. 750 synchronized L1 larvae were transferred into NGM or NGMg plates holding water (control group) or pA1c until the L4 stage. Worms were collected, washed with PBST, and mounted on a 2% agarose pads with a 1% of sodium azide.

### 3.7. Daf-16:GFP Assay

Aged-synchronized L1 larvae were transferred to NGM or NGMg plates previously treated with or without pA1c and incubated for 46 h (until they reached L4 larval stage) at 20 °C. Then, worms were mounted on 2% agarose pads with 1% sodium azide to anesthetize the worms.

### 3.8. Image Acquisition and Quantification

For all conditions tested, approximately 300 animals were fixed and stained. Images of Nile Red, ORO and *daf16:GFP* assays were taken under the same conditions (calibration: 0.68 μm/px; optics: SHR Plan Apo 1×; numerical aperture: 0.15; refractive index: 1; format: 1280 × 960 2 × 2 Binning; exposure: ME 800 ms (−2.0 EV); Analog Gain: 2.00; metering mode: average; BithDepth: 8; zoom: 10×. Fluorescent images of Nile Red stained worms were captured at 10× magnification on a Nikon SMZ18 research stereomicroscope equipped with an epi-fluorescence system and a DS-FI1C refrigerated color digital camera (Nikon Instruments Inc., Tokyo, Japan). Images were taken at the same conditions and integration time under a GFP filter (Ex 480–500; DM 505; BA 535–550). For the ORO analysis, images were also captured at 10× magnification with a Nikon SMZ18 research stereomicroscope equipped with a Nikon DS-Fi2 high-definition color camera. The dihydroethidium (DHE)-labeled ROS formation and the lipofuscin autofluorescence were detected by measuring the fluorescence intensity using a Nikon Eclipse 80i epi-fluorescent microscope, equipped with a TRITC filter (Ex 540–625; DM 565; BA 605–655) and the DAPI filter (with excitation at 340–380 nm and emission at 435–485 nm), respectively (Nikon Instruments Inc., Tokyo, Japan). Regarding the *daf-16* intracellular localization assay, worms were photomicrographed using a GFP (with excitation of 340–380 nm and emission of 435–485 nm) in a Nikon SMZ18 (Nikon Instruments Inc., Tokyo, Japan) fluorescence microscope. In all cases, the image analysis of the Nile Red, ORO, DHE, and lipofuscin assays was performed using ImageJ v1.53e software. The mean value, calculated as the fluorescence mean value per pixel, together with the integrated density and the volume of the worms, were determined. Approximately 25–40 worms were examined in four independent experiments for each condition.

### 3.9. Lifespan Analysis

Synchronized L1 larvae were transferred to NGM or NGMg plates containing water (control group) or pA1c for 46 h, to allow *C. elegans* to develop to L4 stage. Four replicates were used per condition. At least 50–65 L4 larvae per replicate were transferred then onto new plates containing the same treatment that worm has been exposed to +40 μM of 5-fluoro-2-deoxyuridine (FUDR, #856657, Sigma-Aldrich, St. Louis, MO, USA). Surviving or dead animals were counted daily, until all nematodes died. Worms were scored as dead when they failed to respond to gentle touch with a platinum wire.

### 3.10. Egg Lying

To ensure that the treatment with the bacterium did not affect the development of the worm, egg lying was observed in young adult nematodes (day 3 of growth) grown on NGM and NGMg agar plates supplemented or not with the pA1c. The images were taken at 135× magnification using a Nikon SMZ18 stereomicroscope equipped with a Nikon DS-Fi1C high-definition color camera (Nikon Instruments Inc., Tokyo, Japan).

### 3.11. RNA Extraction and a Quantitative PCR Analyses

For gene expression analyses, synchronized L1 wild-type worms were exposed to NGM and NGMg treated with 5 × 10^6^ CFU/mL of pA1c or non-treated NGM and NGMg in eight biological replicates. Trizol^®^ RNA isolation reagent (Thermo Fisher Scientific, Paisley, UK) was used to extracted total RNA from *C. elegans* N2 strain according to the manufacturer’s instructions. NanoDrop ND-1000 spectrophotometer (Thermo Fisher Scientific, Wilmington, DE, USA) was used to determined concentration and purity of RNA at 260/280 nm. Afterwards, 1000 ng of RNA was treated with DNAse (AmbionTM DNase I, RNase-free; Thermo Fisher Scientific Inc., Waltham, MA, USA) according to the manufacturer’s protocol. For the quantitative gene expression analyses, DNA-free RNA was reverse-transcribed into cDNA. Gene expression analyses were performed by quantitative real-time PCR (qPCR) using the TaqMan Universal PCR master mix and specific probes from Applied Biosystems Technologies (Thermo Fisher Scientific Inc., Waltham, MA, USA) and Integrated DNA Technologies Inc. (Coralville, IA, USA). All reactions were performed using a CFX384 TouchTM Real-Time PCR Detection System (BioRad, Hercules, CA, USA). The expression level of each gene was normalized compared to the expression of the *pmp-3* gene from Life Technologies (TaqMan Gene Expression Assays, Carlsbad, CA, USA), which were used as housekeeping gene control. Gene expression differences between treated and untreated worms were quantified using the relative quantification 2^−∆∆Ct^ method.

### 3.12. Statistical Analysis

*C. elegans* body fat reduction (Nile Red and Oil Red O) between treatment and control condition (NGM group), together with oxidative stress (DHE), lipofuscin determinations, and real-time PCR data were evaluated by a 2 × 2 ANOVA test (pA1c and glucose), followed by multiple comparison (Student’s *t*-test) tests. For lifespan assays, log-rank (Mantel-Cox test) between pA1c and control (NGM) treatments were performed. All tests were performed using StataSE v14 software (StataCorp LLC, College Station, TX, USA).

## 4. Conclusions

In conclusion, we have shown that the strain of *Pediococcus* *acidilactici* CECT9879 (pA1c) reduces fat accumulation in *C. elegans*. Moreover, we have demonstrated that the molecular mechanisms by which this strain exerts this fat-reducing effect are modulating the IIS (reverting the *daf-16*-nuclear-translocation effect of high-glucose and returning *daf-16* into the cytosol), inhibiting the FA biosynthesis, and activating mitochondrial and peroxisomal FA degradation. In addition, supplementation with pA1c enhanced oxidative stress response in a high-glucose condition by reducing ROS levels through SKN-1/Nrf2 mediation, improving aging in a glucose-loaded (10 mM) NGM medium and increasing lifespan in *C. elegans* by affecting DAF-16/FOXO (overexpressing *daf-16* and *ins-6*, and inhibiting *daf-2*) and impairing the IIS and NHR-49/PPAR-α metabolic pathways.

Taken together, our data suggest that supplementation with pA1c significantly improves the response of carbohydrate and lipid metabolism in *C. elegans*. Hence, pA1c could be considered a potential probiotic strain for the prevention of the metabolic syndrome-related disturbances, such as type-2 diabetes and obesity, and highlights the use of *C. elegans* as an appropriate in vivo model for study of the mechanisms underlying these diseases.

## 5. Patents

Part of the results that serve as the basis for this manuscript have been included in the patent entitled: Probiotics for regulating blood glucose [PCT/EP2020/087284; WO2021123355A1].
